# Facial Fractures Associated With Craniomaxillofacial Trauma in Adults: A Literature Review

**DOI:** 10.7759/cureus.90382

**Published:** 2025-08-18

**Authors:** Freddy Lizano Guevara, David Sáenz Araya, Santiago Daniel Baizan Orias, Enmanuel Sevilla Torres, Alberto Rojas Peláez, Daniela Fernandez Vinocour

**Affiliations:** 1 General Medicine, Universidad de Ciencias Médicas (UCIMED), San José, CRI

**Keywords:** bones, facial bones, facial fractures, fractures, maxillofacial injuries, reconstructive surgical procedures, wounds and trauma

## Abstract

Craniomaxillofacial trauma includes all injuries to the head, face, and jaw. Among these, facial fractures are particularly significant because of their functional, aesthetic, and emotional impact. They are most frequently sustained by young men and can affect essential functions such as chewing, speaking, and breathing, while also leading to psychological consequences. The purpose of this review was to examine recent scientific literature on facial fractures within the context of craniomaxillofacial trauma, focusing on epidemiology, diagnosis, and management.

A bibliographic search was conducted in PubMed, Scopus, and ScienceDirect for articles published between 2020 and 2025, in Spanish and English. The inclusion criteria were original publications, systematic reviews, and evidence-based clinical guidelines. Exclusion criteria included duplicates, studies limited to pediatric populations, and those with low clinical applicability. The review incorporated observational studies, narrative reviews, and publications from scientific societies. The findings showed that the most common fracture types were nasal, mandibular, and zygomatic complex fractures. The main causes were interpersonal violence, traffic accidents, and falls, with the latter more frequent among older adults. Computed tomography (CT) scanning remains the imaging modality of choice. Advances in surgical techniques, including virtual planning, intraoperative navigation, and 3D printing, have improved accuracy and reduced complications. Nevertheless, challenges such as malocclusion, facial asymmetry, and postoperative infection persist.

The management of facial fractures requires a multidisciplinary, structured approach supported by scientific evidence and technological innovation. Continued improvement in both functional and aesthetic outcomes depends on strengthening professional training, advancing high-quality clinical research, and ensuring equitable access to specialized care.

## Introduction and background

Craniomaxillofacial trauma refers to injuries involving the head, face, and jaw, which present unique clinical challenges due to their complexity and significant impact on both function and appearance [1]. 

Globally, facial fractures are a significant public health concern. In 2019, about 8.9 million new cases were reported worldwide, contributing substantially to years lived with disability [1]. Epidemiological data indicate that young men aged 20-29 are the most affected, with a male-to-female ratio of at least 3:1 [2,3]. Facial fractures are particularly important clinically because they can impair essential functions such as chewing, speaking, and breathing, while also carrying profound psychological consequences, especially in younger patients [4]. The main causes include falls and traffic accidents, especially in urban areas or regions with limited preventive measures. These injuries often require specialized multidisciplinary care, extended rehabilitation, and impose considerable financial strain on healthcare systems [1].

Despite advances in both understanding and management, a comprehensive review is still needed to synthesize current evidence on the clinical presentation, epidemiology, and therapeutic approaches to facial fractures within the broader scope of craniomaxillofacial trauma.

This review therefore aims to provide an updated, evidence-based overview of facial fractures, with emphasis on their clinical, epidemiological, and therapeutic implications.

## Review

Methods

The present review was conducted using a directed yet flexible search strategy to capture the current state of knowledge on facial fractures within the context of craniomaxillofacial trauma. Searches were performed in three major biomedical databases: PubMed, Scopus, and ScienceDirect. Priority was given to publications from 2020 to 2025, in both English and Spanish. The search strategy combined descriptors such as maxillofacial injuries, facial bones, fractures, bones, wounds and injuries, and reconstructive surgical procedures, refined with Boolean operators to improve clinical and therapeutic relevance.

A selective approach was applied, prioritizing clinical applicability, recency of findings, and methodological strength rather than adhering to a rigid framework. Eligible studies included original research articles, systematic reviews, and expert consensus statements from recognized scientific societies. Exclusion criteria included duplicates, studies with limited clinical applicability, the absence of relevant statistical analysis, and those focusing primarily on pediatric populations.

**Figure 1:**
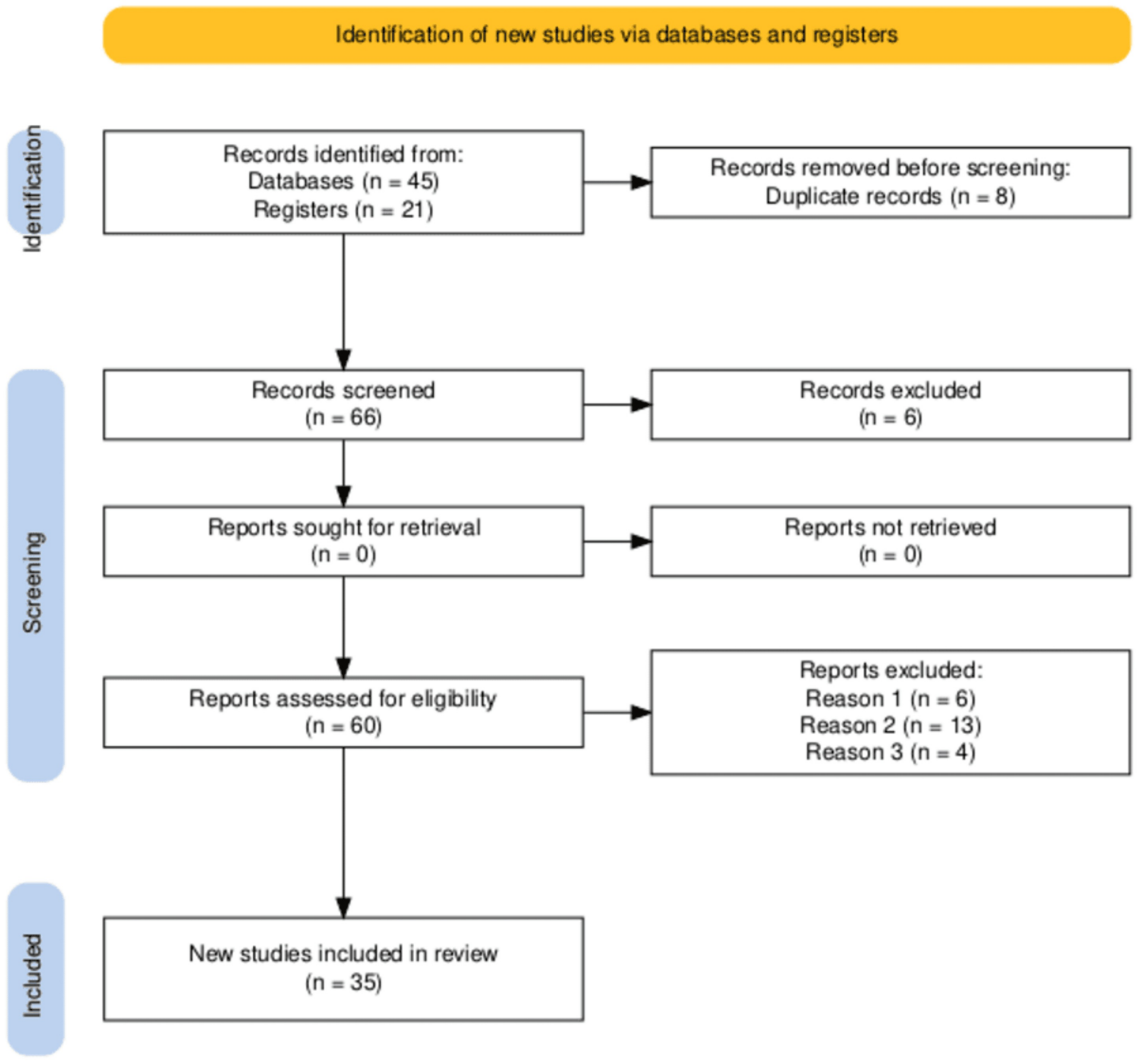
PRISMA flow diagram of the study selection process Reason 1: absence of statistical analysis/significance, Reason 2: findings not applicable to clinical settings, Reason 3: pediatric studies. Diagram created using the PRISMA flow diagram tool [5].

From an initial set of publications, 66 articles were identified, with 8 duplicates removed. After screening and applying the exclusion criteria, a final sample of 35 studies was included in the review (Figure 1).

The final corpus was composed mainly of broad-spectrum observational studies, well-structured narrative reviews, multicenter epidemiological analyses, and evidence-based clinical practice documents. This mix allowed for a comprehensive and critical perspective on the topic, combining quantitative evidence with the clinical-practical insights needed for the holistic management of these injuries.

It should also be noted that artificial intelligence was used as a complementary tool in preparing this manuscript. Its role was to support structure, synthesis, and coherence of the text, without replacing the authors’ critical analysis or academic judgment.

Epidemiology and etiology

Maxillofacial fractures are most prevalent in young adults, particularly males between 20 and 29 years of age. Reported male-to-female ratios range from 3.3:1 to as high as 9.6:1 [2,4]. The most commonly affected sites are the nasal bone, mandible, and zygomatic complex, with the nasal bone frequently cited as the most common site across different regions [6]. While the overall number of maxillofacial fractures has increased globally, age-standardized fracture rates have declined in some regions, likely reflecting improvements in safety protocols or demographic variations [1,7].

The primary mechanisms of injury include interpersonal violence, traffic accidents, and falls. Violence accounts for a large proportion of cases in certain regions [2,6], while traffic-related injuries are most frequent in areas with high vehicle density [4,7]. In older adults, falls are the leading cause of maxillofacial fractures [1,8]. Key risk factors include male sex, young age, alcohol consumption, and low educational attainment [2,4]. Urban environments are associated with higher rates of violence-related injuries, whereas rural settings more commonly involve domestic and occupational accidents. The use of safety devices such as seatbelts and airbags significantly reduces the severity of injuries, particularly in rollover crashes [8].

Classification of facial fractures

Nasal fractures are the most common injuries of the facial skeleton, owing to their prominent location and fragile structure. The nasal bones form the bony nasal pyramid and articulate with the frontal and maxillary bones [9,10]. Depending on the severity and extent, nasal fractures are classified into three types: Type I fractures are low-impact and do not involve the nasal septum; Type II fractures extend to the septum and anterior nasal spine; and Type III fractures involve the orbital structures, nasal septum, and, in severe cases, may extend intracranially [10].

Orbital fractures also represent a significant proportion of craniomaxillofacial injuries, with some trauma centers reporting rates as high as 41.4%. These fractures are often associated with falls and may lead to functional and aesthetic complications. Common sequelae include diplopia, caused by entrapment of the extraocular muscles, and enophthalmos, resulting from loss of orbital volume [9,10].

Zygomatic fractures, particularly those involving the zygomaticomaxillary complex, are clinically significant due to their impact on facial symmetry, contour, and proximity to the orbital rim. These injuries can compromise both aesthetics and function, making accurate anatomical reduction and stable fixation critical for optimal outcomes. Intraoperative cone-beam computed tomography (CBCT) has been shown to enhance surgical precision by allowing real-time assessment and correction of bone alignment during intervention [11].

Maxillary fractures are classically described using the Le Fort classification, which defines three main variants: Le Fort I, II, and III. Le Fort I (transmaxillary fracture) is a horizontal fracture of the lower maxilla involving the hard palate and alveolar processes. On imaging, it typically includes the pterygoid plates and lateral walls of the maxillary sinuses, with disruption of the anterolateral border of the nasal fossa [10]. Le Fort II (pyramidal fracture) is characterized by the separation of the maxilla and nasal bones from the skull base. The fracture line usually involves the nasal bones, frontal process of the maxilla, lacrimal bone, orbital floor, and often the pterygoid plates. Radiologically, discontinuity of the infraorbital rim is a common finding [10]. Lastly, Le Fort III (craniofacial disjunction) is the most severe type, representing complete separation of the facial skeleton from the cranium. The fracture line extends through the nasofrontal suture to the ethmoid and sphenoid bones and involves the frontal process of the zygoma, lateral orbital wall, zygomatic arch, and pterygoid plates. Clinically, disruption of the zygomatic arch is often considered a key diagnostic feature [10].

Mandibular fractures are the second most common facial fractures after nasal bone fractures. Owing to its U-shape and articulation with the skull at the temporomandibular joints, the mandible functions as a bony ring. This structural configuration predisposes patients to contralateral or “mirror” fractures, making comprehensive examination essential for accurate diagnosis and treatment planning [10].

Frontal bone fractures may extend into the frontal sinus. Involvement of the posterior wall, whether isolated or in combination, carries a high risk of cerebrospinal fluid leak and intracranial complications. Accurate characterization of these fractures relies heavily on advanced imaging, which guides surgical planning and repair [10].

Panfacial fractures, defined as involvement of multiple facial subunits, represent one of the most challenging scenarios in craniomaxillofacial trauma. Their management requires a multidisciplinary and stepwise approach to restore both function and aesthetics. The Facial Injury Severity Scale (FISS) has been validated as a tool to stratify severity, assist in treatment planning, and facilitate communication among surgical teams [12].

Diagnosis

The clinical assessment of craniomaxillofacial trauma should begin with a primary survey aimed at stabilizing the patient, in accordance with Advanced Trauma Life Support (ATLS) principles. This includes securing the airway, controlling bleeding, and performing a thorough, systematic evaluation to avoid missed injuries [13,14].

Key clinical signs to assess include local tenderness, ecchymosis, swelling, deformity, malocclusion, pain on palpation, and restricted mouth opening. Incorporating standardized screening checklists, which account for intraoral findings, can improve consistency and accuracy during examination [14].

**Figure 2:**
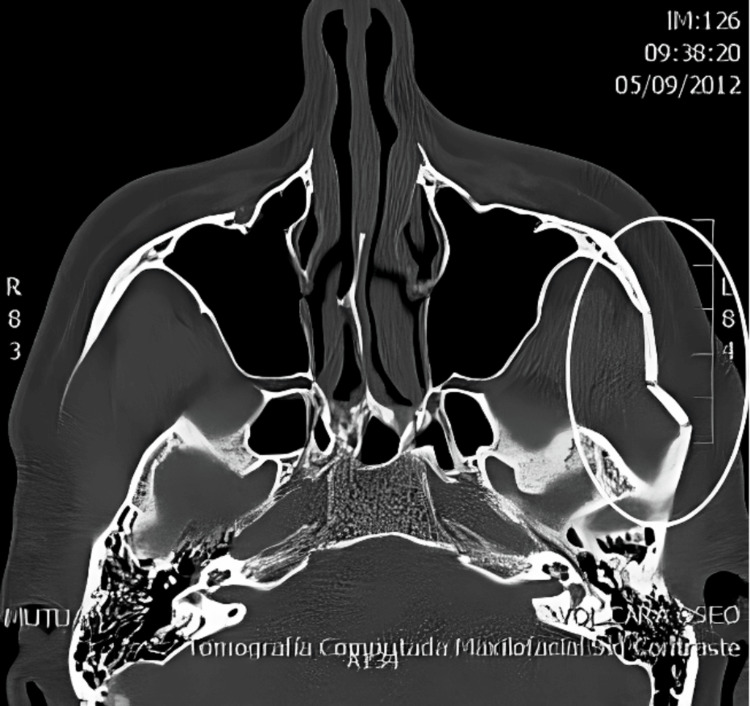
Preoperative non-contrast axial CT scan showing an “M”-shaped left zygomaticomaxillary complex fracture Reproduced from reference [16] (Creative Commons Attribution-NonCommercial-NoDerivatives 4.0 (CC BY-NC-ND 4.0)).

Plain radiography, while commonly used as an initial modality, has significant limitations in evaluating complex injuries. CT has become the gold standard for diagnosis, offering detailed three-dimensional visualization of fracture location, morphology, and associated injuries (Figure 2). CT is particularly critical in cases of multiple fractures or high-energy trauma, such as motor vehicle accidents [13,15].

Magnetic resonance imaging (MRI) is useful for soft tissue assessment and suspected nerve injuries. Ultrasound can aid in evaluating superficial structures, though its role in assessing bony injuries is limited. In children, facial fractures are uncommon but require special consideration due to ongoing facial growth. While non-operative management is often preferred, precise reduction of displaced fractures is essential to prevent long-term deformity [17,18].

Principles of therapeutic management

The ATLS protocol is essential in managing facial trauma, particularly for airway protection, hemorrhage control, and structured evaluation to avoid missed injuries [14,18]. Airway management is especially important in cases of midface fractures or cranial trauma due to the risk of obstruction. Once the patient is stabilized, definitive treatment decisions rely on diagnostic imaging [13,18].

In polytrauma patients, a multidisciplinary approach within the "golden hour" of in-hospital care is critical for favorable outcomes [19]. The most common emergency procedures performed during this period include laparotomy and chest tube placement, while fracture fixation is usually delayed and guided by the "safe definitive surgery" principle [20]. 

Surgical fixation is indicated for displaced fractures or those unsuitable for conservative treatment to prevent permanent deformity. Conservative management remains more common, especially in children, unless significant displacement is present [18,19]. Management decisions take into account factors such as the number of fractures, mechanism of injury, and the patient’s age and sex [8]. Emergency priorities focus on airway compromise, major hemorrhage, and traumatic brain injury. Overall, access to specialized expertise plays a key role in improving outcomes in complex cases [20].

Update on surgical treatment

Metal plates and screws remain the dominant osteosynthesis technique due to their strength and reliability, especially in complex fractures such as those of the zygomaticomaxillary complex where stability is critical [10]. Resorbable systems are increasingly used in pediatric patients, as they provide adequate stability without interfering with growth and carry a lower risk of long-term complications [21,22].

**Figure 3:**
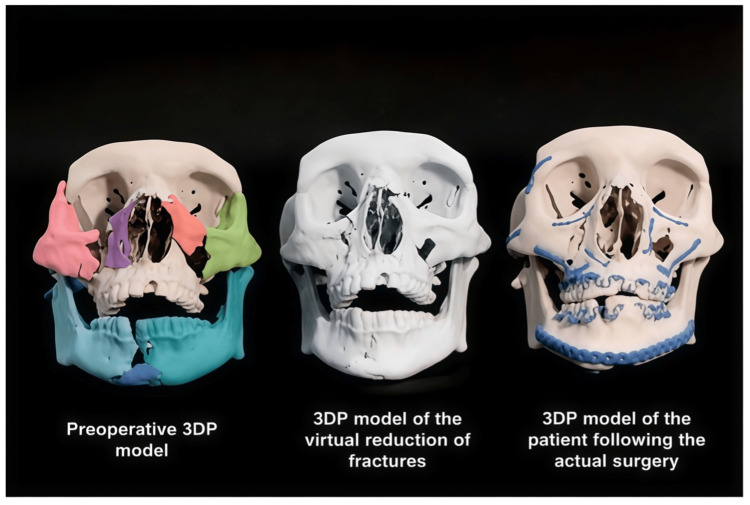
Sequential patient-specific 3D-printed models from the EPPOCRATIS protocol Reproduced from reference [21] (Creative Commons Attribution 4.0 (CC BY 4.0)).

Recent advances, including virtual surgical planning and 3D printing, have improved the precision of complex reconstructions. The EPPOCRATIS system is one example, showing favorable outcomes in acute facial trauma [21] (Figure 3). For subcondylar fractures, endoscopic approaches may enhance reduction accuracy while reducing the risk of facial nerve injury [23].

Management is specific to the type of fracture. Mandibular fractures are usually treated with open reduction and internal fixation to restore masticatory function. Compared with traditional dental splints, intermaxillary fixation screws provide advantages such as greater patient comfort and reduced operative time [24]. For zygomaticomaxillary fractures, intraoperative imaging may be necessary to ensure proper alignment, while navigation systems or custom implants can further improve stability [11].

Anesthetic care is case-specific, with particular attention to airway safety. In fractures involving the skull base or causing airway obstruction, conventional orotracheal intubation may be unsafe. Passing the tube through a disrupted cranial base risks intracranial injury; therefore, a tracheostomy or cricothyrotomy may be required [25].

3D imaging is now the standard for evaluating orbital deformities and planning treatment, as 2D imaging often proves inadequate. CBCT or helical CT scans are preferred for bony structures, given their lower error rates in assessing shape and measurements. For soft tissue complications, such as muscle entrapment or infection, contrast-enhanced CT or MRI is more appropriate. The digital workflow typically begins with exporting DICOM data into planning software for analysis. Examples include Voxim® (IVS Solutions AG, Chemnitz, Germany) and Elements® (Brainlab, Munich, Germany), which allow virtual modeling and STL export for custom implant design. Trumatch® (DePuy Synthes, West Chester, PA, USA) is particularly useful for integrating design and manufacturing in a streamlined workflow [26].

Patient-specific implants are designed to be thin, stable, and anatomical, relying on guided trajectories or landmarks for intraoperative navigation. Selective laser melting titanium is the favored manufacturing method, offering strength, radiopacity, and adaptability for complex geometries. Additional contours or flanges may be incorporated to assist with positioning and stabilization. Designs can be directly 3D-printed or used to create physical biomodels for intraoperative reference [26].

Intraoperative navigation has further enhanced surgical precision by enabling real-time verification and adjustment of implant placement. Both pointer-based and trajectory-guided navigation, combined with intraoperative imaging such as CBCT, allow same-day modifications when needed. Newer navigation platforms also include dynamic components, improving the accuracy of complex orbital reconstructions and reducing complication rates [26].

Complications and follow-up

Postoperative infectious complications are common, whether in aesthetic procedures or surgeries involving bone grafts or implants. The risk of infection depends on the patient's health status and the complexity of the procedure. Another frequent complication is malocclusion, often caused by inadequate fracture reduction or incomplete restoration of occlusion. These cases may require further surgical intervention, including orthognathic surgery followed by orthodontic treatment [27].

Facial asymmetry is another complication, particularly in zygomaticomaxillary fractures where insufficient reduction or fixation of the zygomatic bone occurs, often necessitating secondary correction [11,28]. Neurological injury from the initial trauma can also result in long-term sensory or motor deficits [11]. To reduce these risks, advanced techniques such as computer-assisted surgical planning, 3D-printed plates, and intraoperative navigation have been adopted, especially for complex fractures such as those involving the skull base [11]. These methods improve surgical precision, reduce malocclusion and asymmetry, and enhance predictability. The use of autologous mesenchymal stem cells has also emerged as an alternative to grafting materials, offering better osseointegration and reduced risk of rejection [29].

Functional and aesthetic rehabilitation typically incorporates prosthetic restoration through dental implants, where the success of the implant has the proclivity to be impacted by radiation therapy history or other systemic conditions [30,31]. When interference, asymmetry, malocclusion, or functional deficiencies persist, secondary interventions may be required. These may include zygomatic repositioning osteotomy with bone grafting [28] or combined procedures such as Le Fort I osteotomy with implant placement, which restore function while improving aesthetics [30].

Indications for secondary correction include persistent deformities, orbital asymmetry, or malpositioned bone segments causing significant aesthetic or functional problems [30]. Similarly, malpositioned or nonfunctional implants may require surgical revision to restore occlusion [27,32]. For patients dissatisfied with cosmetic outcomes, final revision surgery may be considered, with improvement in facial appearance as the primary goal [28].

Multidisciplinary approaches

Effective management of craniomaxillofacial trauma requires a multidisciplinary approach, with the maxillofacial surgeon playing a central role. Maxillofacial surgery addresses fracture reduction, bone reconstruction, soft tissue repositioning, and restoration of both function and aesthetics, while also aiming to prevent complications that may accelerate facial aging [14].

Otolaryngology is often involved, given the high frequency of injuries to the nose and paranasal sinuses, and works in close collaboration with maxillofacial surgery to manage the broader head and neck structures [33]. Depending on the severity of the injury, ophthalmology may also be critical, allowing early assessment and management of ocular injuries with a focus on vision preservation and orbital integrity [14].

When intracranial injury is suspected, neurosurgery becomes essential due to the close anatomical relationship between the brain and facial skeleton. In addition, rehabilitation teams support functional recovery and reintegration, although evidence on their overall effectiveness remains variable [34].

Beyond physical reconstruction, facial trauma carries a significant psychological burden, as changes in appearance and function can trigger anxiety, depression, or post-traumatic stress. Psychological support is therefore a key component of recovery. Tools such as the AO-CMF Patient-Reported Outcome Battery have been developed to better capture patient concerns and guide targeted psychological interventions [35].

Discussion

Particularly, a key factor in current clinical practice is the issue of interfacility transfers. Efforts have been made to reduce unnecessary referrals of mild cases to tertiary centers, in order to optimize resource use and streamline care [33]. Likewise, prioritizing stabilization during the primary survey before initiating diagnostic or surgical interventions remains a central principle, particularly in patients with polytrauma [13].

Complex fractures, such as panfacial injuries, continue to present major therapeutic challenges. Standardized surgical planning is critical, often requiring staged procedures and close attention to airway management. In such cases, alternative techniques like submental intubation have emerged as valuable options to reduce the risks associated with tracheostomy [36].

Despite these advances, significant gaps remain in the literature. Many treatment decisions are still based on expert opinion and retrospective studies, highlighting the need for more high-quality prospective research [13]. Similarly, while new technologies have shown promise, they are not yet routinely integrated into clinical practice and require stronger scientific validation as well as broader, equitable access [21]. In this context, improving the training of healthcare providers, particularly in resource-limited settings, and developing structured protocols that cover the entire continuum, from initial stabilization to final rehabilitation, remain essential priorities [14].

## Conclusions

Facial fractures are a significant clinical and public health problem due to their functional, aesthetic, and psychosocial consequences, particularly in younger men affected by violence, vehicular accidents, and falls. They occur with notable frequency and complexity, underscoring the need for focused preventive strategies and comprehensive multidisciplinary management. Despite many advancements in diagnosis and treatment, surgical and postsurgical complications, such as malocclusion, asymmetry, and neurological sequelae, remain common and often require long-term follow-up or secondary interventions.

Advances in technology, including CT, virtual surgical planning, intraoperative navigation, and customized 3D-printed implants, have improved surgical accuracy and safety. However, effective outcomes depend not only on access to these tools but also on a systemic approach that incorporates standardized protocols, team-based care, comprehensive training, and equitable resource distribution. Together, these measures support optimal functional and psychosocial recovery, especially for patients with complex needs that go beyond standard approaches, even in the most resource-rich settings.
